# 6-Ethyl-5-fluoro-2-methoxy­pyrimidin-4(3*H*)-one

**DOI:** 10.1107/S1600536809035430

**Published:** 2009-09-30

**Authors:** Yu-Yuan Ye, Kai Yang

**Affiliations:** a School of Life Science and Pharmaceutical and Chemical Engineering, Taizhou University, Linhai 317000, People’s Republic of China; b College of Biological and Environmental Engineering, Zhejiang University of Technology, Hangzhou 310014, People’s Republic of China

## Abstract

In the title compound, C_7_H_9_FN_2_O_2_, the meth­oxy and ethyl groups form dihedral angles of 1.4 (2) and 73.5 (3)°, respectively, with the mean plane of the pyrimidine ring. In the crystal structure, two mol­ecules are linked by a pair of N—H⋯O hydrogen bonds, forming a centrosymmetric dimer.

## Related literature

For fluoro-containing pyrimidines as inter­mediates for the synthesis of some anti­cancer and anti­fungal drugs, see: Bergmann *et al.* (1959 ▶); Butters *et al.* (2001 ▶).
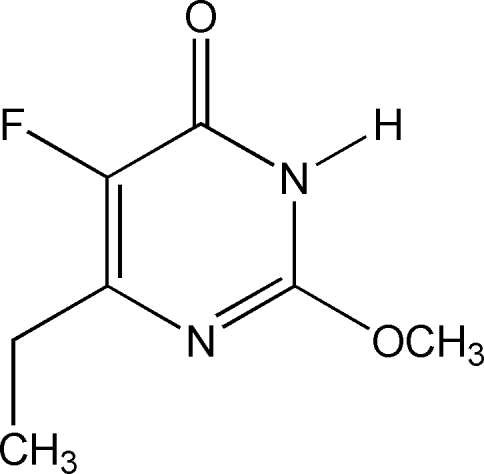


## Experimental

### 

#### Crystal data

C_7_H_9_FN_2_O_2_
*M* *_r_* = 172.16
Triclinic, 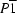

*a* = 4.5711 (4) Å
*b* = 8.4985 (8) Å
*c* = 10.8546 (11) Å
α = 88.043 (2)°
β = 79.737 (3)°
γ = 79.616 (2)°
*V* = 408.13 (7) Å^3^
*Z* = 2
Mo *K*α radiation
μ = 0.12 mm^−1^
*T* = 296 K
0.40 × 0.28 × 0.18 mm

#### Data collection

Rigaku R-AXIS RAPID diffractometer
Absorption correction: multi-scan (**ABSCOR**; Higashi, 1995 ▶) *T*_min_ = 0.948, *T*_max_ = 0.979
4010 measured reflections
1842 independent reflections
945 reflections with *I* > 2σ(*I*)
*R*_int_ = 0.019

#### Refinement

*R*[*F* ^2^ > 2σ(*F* ^2^)] = 0.047
*wR*(*F* ^2^) = 0.106
*S* = 1.00
1842 reflections
111 parameters
H-atom parameters constrained
Δρ_max_ = 0.39 e Å^−3^
Δρ_min_ = −0.37 e Å^−3^

### 

Data collection: *PROCESS-AUTO* (Rigaku/MSC, 2004 ▶); cell refinement: *PROCESS-AUTO*; data reduction: *CrystalStructure* (Rigaku/MSC, 2004 ▶); program(s) used to solve structure: *SHELXS97* (Sheldrick, 2008 ▶); program(s) used to refine structure: *SHELXL97* (Sheldrick, 2008 ▶); molecular graphics: *ORTEP-3* (Farrugia, 1997 ▶); software used to prepare material for publication: *CrystalStructure* (Rigaku/MSC, 2004 ▶).

## Supplementary Material

Crystal structure: contains datablocks global, I. DOI: 10.1107/S1600536809035430/is2454sup1.cif

Structure factors: contains datablocks I. DOI: 10.1107/S1600536809035430/is2454Isup2.hkl

Additional supplementary materials:  crystallographic information; 3D view; checkCIF report

## Figures and Tables

**Table 1:** Hydrogen-bond geometry (Å, °)

*D*—H⋯*A*	*D*—H	H⋯*A*	*D*⋯*A*	*D*—H⋯*A*
N1—H1⋯O2^i^	0.86	1.91	2.763 (2)	174

Symmetry code: (i) 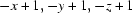.

**Fig. 1.**
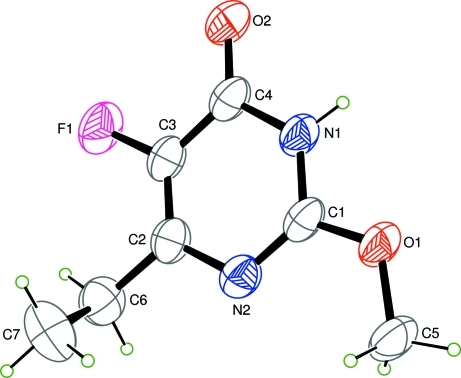
The molecular structure of (I) with 30% probability displacement ellipsoids.

**Fig. 2.**
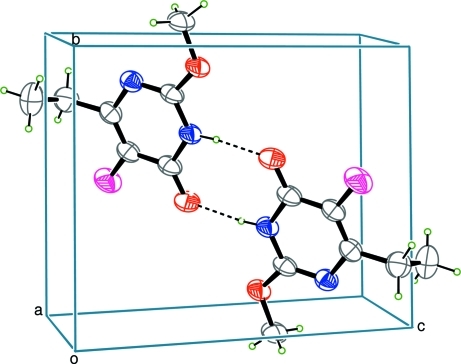
Packing diagram of (I), showing hydrogen bonds as dashed lines.

## supplementary crystallographic information

### Comment

The fluoro-containing pyrimidines have been used as a kind of important intermediates for the synthesis of some anticancer drugs and antifungal drugs (Bergmann *et al.*, 1959; Butters *et al.*, 2001). In the synthesis of the novel antifungal drug-Voriconazole, we have prepared the title compound 6-ethyl-5-fluoro-2-methoxypyrimidin-4(3*H*)-one as an intermediate, which was synthesized by reacting methyl 2-fluoro-3-oxopentanoate with *o*-methylisourea sulfate in a solution of sodium methylate in methanol.

The molecular structure of the title compound, (I), is illustrated in Fig. 1. The bond lenghth of C4—O2 and C1—O1 are 1.238 (3) and 1.321 (2) Å, respectively, corresponding to a double C=O bond and a C*sp*^2^—O single bond. In the six-membered pyrimidine ring, the even bond lengths of C—N and C—C are 1.361 (3) and 1.380 (3) Å, respectively, indicating these bond forming a conjugating system. The atoms in the pyrimidine ring (C1–C4/N1/N2) form a good plane with a mean deviation of 0.006 Å. An intermolecular N—H···O hydrogen bond was found to link two molecules as a pair (Fig. 2 and Table 1).

### Experimental

To a 250 ml flask was added a 80 ml solution of 25% sodium methylate in methanol. The solution was cooled to 278 K, and then 40 g *o*-methylisourea sulfate and 20 g methyl 2-fluoro-3-oxopentanoate were added. After the addition, the mixture were stirred at 298 K for half an hour and refluxed for three hours. The mixture was concentrated under reduced pressure, and the residue was dissolved with 200 ml water. The aqueous solution was treated with 6*M* hydrochloric acid to pH3 and cooled in refrigerator for three hours. The resulted precipitate was filtered, to give 12.5 g product as white powder (yield 53.8%; m.p. 447–449 K). Since the product was not found to be suitable for X-ray diffraction studies, a few samples were dissolved in absolute ethanol, which was allowed to evaporate slowly to give colourless crystals of (I) suitable for X-ray diffraction studies.

### Refinement

H atoms were placed in calculated positions (C—H = 0.96–0.97 Å and N—H = 0.86 Å) and refined using a riding model, with *U*_iso_(H) = 1.2*U*_eq_(C, N) or 1.5*U*_eq_(methyl C).

### Crystal data

**Table:**

C_7_H_9_FN_2_O_2_	*Z* = 2
*M**_r_* = 172.16	*F*(000) = 180.00
Triclinic, *P*1	*D*_x_ = 1.401 Mg m^−^^3^
Hall symbol: -P 1	Mo *K*α radiation, λ = 0.71075 Å
*a* = 4.5711 (4) Å	Cell parameters from 2411 reflections
*b* = 8.4985 (8) Å	θ = 3.1–27.4°
*c* = 10.8546 (11) Å	µ = 0.12 mm^−^^1^
α = 88.043 (2)°	*T* = 296 K
β = 79.737 (3)°	Chunk, colorless
γ = 79.616 (2)°	0.40 × 0.28 × 0.18 mm
*V* = 408.13 (7) Å^3^	

### Data collection

**Table:**

Rigaku R-AXIS RAPID diffractometer	945 reflections with *I* > 2σ(*I*)
Detector resolution: 10.00 pixels mm^-1^	*R*_int_ = 0.019
ω scans	θ_max_ = 27.4°
Absorption correction: multi-scan (*ABSCOR*; Higashi, 1995)	*h* = −5→5
*T*_min_ = 0.948, *T*_max_ = 0.979	*k* = −10→11
4010 measured reflections	*l* = −14→14
1842 independent reflections	

### Refinement

**Table:**

Refinement on *F*^2^	*w* = 1/[σ^2^(*F*_o_^2^) + (0.*P*)^2^ + 0.345*P*] where *P* = (*F*_o_^2^ + 2*F*_c_^2^)/3
*R*[*F*^2^ > 2σ(*F*^2^)] = 0.047	(Δ/σ)_max_ < 0.001
*wR*(*F*^2^) = 0.106	Δρ_max_ = 0.39 e Å^−^^3^
*S* = 1.00	Δρ_min_ = −0.37 e Å^−^^3^
1842 reflections	Extinction correction: *SHELXL97* (Sheldrick, 2008)
111 parameters	Extinction coefficient: 0.025 (2)
H-atom parameters constrained	

### Special details

**Table:**

Geometry. All e.s.d.'s (except the e.s.d. in the dihedral angle between two l.s. planes) are estimated using the full covariance matrix. The cell e.s.d.'s are taken into account individually in the estimation of e.s.d.'s in distances, angles and torsion angles; correlations between e.s.d.'s in cell parameters are only used when they are defined by crystal symmetry. An approximate (isotropic) treatment of cell e.s.d.'s is used for estimating e.s.d.'s involving l.s. planes.
Refinement. Refinement using all reflections. The weighted *R*-factor (*wR*) and goodness of fit (*S*) are based on *F*^2^. *R*-factor (gt) are based on *F*. The threshold expression of *F*^2^ > 2.0 σ(*F*^2^) is used only for calculating *R*-factor (gt).

### Fractional atomic coordinates and isotropic or equivalent isotropic displacement parameters (Å^2^)

**Table:**

	*x*	*y*	*z*	*U*_iso_*/*U*_eq_	
F1	−0.2750 (3)	0.5521 (2)	0.78974 (17)	0.0840 (5)	
O1	0.6346 (4)	0.0891 (2)	0.60826 (17)	0.0674 (5)	
O2	0.1625 (4)	0.6002 (2)	0.58950 (19)	0.0754 (6)	
N1	0.3901 (4)	0.3399 (2)	0.6042 (2)	0.0577 (5)	
N2	0.2058 (4)	0.1623 (2)	0.7615 (2)	0.0587 (5)	
C1	0.4039 (5)	0.1970 (2)	0.6603 (2)	0.0558 (6)	
C2	−0.0212 (5)	0.2877 (3)	0.8040 (2)	0.0579 (6)	
C3	−0.0446 (5)	0.4310 (3)	0.7475 (2)	0.0590 (7)	
C4	0.1656 (6)	0.4689 (3)	0.6427 (2)	0.0603 (7)	
C5	0.6683 (7)	−0.0711 (2)	0.6608 (2)	0.0788 (9)	
C6	−0.2321 (6)	0.2513 (3)	0.9191 (2)	0.0753 (8)	
C7	−0.0903 (8)	0.2402 (4)	1.0339 (2)	0.0997 (11)	
H1	0.5276	0.3520	0.5412	0.069*	
H51	0.7001	−0.0670	0.7457	0.095*	
H52	0.8384	−0.1380	0.6122	0.095*	
H53	0.4886	−0.1142	0.6595	0.095*	
H61	−0.2930	0.1500	0.9075	0.090*	
H62	−0.4085	0.3355	0.9311	0.090*	
H71	0.0851	0.1573	1.0231	0.120*	
H72	−0.2328	0.2156	1.1050	0.120*	
H73	−0.0324	0.3405	1.0473	0.120*	

### Atomic displacement parameters (Å^2^)

**Table:**

	*U*^11^	*U*^22^	*U*^33^	*U*^12^	*U*^13^	*U*^23^
F1	0.0660 (10)	0.0684 (10)	0.1026 (13)	0.0173 (8)	−0.0007 (9)	−0.0156 (9)
O1	0.0774 (13)	0.0441 (9)	0.0690 (12)	0.0153 (9)	−0.0074 (10)	−0.0028 (8)
O2	0.0822 (14)	0.0447 (10)	0.0864 (14)	0.0146 (9)	−0.0062 (11)	0.0003 (9)
N1	0.0625 (13)	0.0437 (11)	0.0595 (13)	0.0091 (10)	−0.0095 (10)	−0.0023 (10)
N2	0.0594 (13)	0.0517 (12)	0.0629 (14)	−0.0013 (10)	−0.0131 (11)	−0.0034 (10)
C1	0.0606 (16)	0.0431 (13)	0.0625 (16)	0.0074 (11)	−0.0230 (13)	−0.0094 (12)
C2	0.0502 (15)	0.0603 (16)	0.0623 (16)	−0.0031 (12)	−0.0122 (12)	−0.0101 (13)
C3	0.0499 (15)	0.0520 (15)	0.0694 (17)	0.0079 (12)	−0.0097 (13)	−0.0117 (13)
C4	0.0620 (17)	0.0456 (14)	0.0698 (17)	0.0082 (12)	−0.0187 (14)	−0.0083 (13)
C5	0.102 (2)	0.0428 (14)	0.082 (2)	0.0155 (15)	−0.0193 (18)	−0.0009 (14)
C6	0.0602 (18)	0.077 (2)	0.085 (2)	−0.0112 (15)	−0.0033 (16)	−0.0051 (17)
C7	0.091 (2)	0.136 (3)	0.069 (2)	−0.029 (2)	0.0017 (18)	0.004 (2)

### Geometric parameters (Å, °)

**Table:**

F1—C3	1.359 (2)	C6—C7	1.496 (4)
O1—C1	1.321 (2)	N1—H1	0.860
O1—C5	1.451 (3)	C5—H51	0.960
O2—C4	1.238 (3)	C5—H52	0.960
N1—C1	1.336 (3)	C5—H53	0.960
N1—C4	1.379 (3)	C6—H61	0.970
N2—C1	1.354 (3)	C6—H62	0.970
N2—C2	1.375 (3)	C7—H71	0.960
C2—C3	1.340 (3)	C7—H72	0.960
C2—C6	1.496 (3)	C7—H73	0.960
C3—C4	1.420 (3)		
			
C1—O1—C5	118.15 (19)	O1—C5—H51	109.5
C1—N1—C4	123.1 (2)	O1—C5—H52	109.5
C1—N2—C2	114.5 (2)	O1—C5—H53	109.5
O1—C1—N1	113.63 (19)	H51—C5—H52	109.5
O1—C1—N2	121.8 (2)	H51—C5—H53	109.5
N1—C1—N2	124.6 (2)	H52—C5—H53	109.5
N2—C2—C3	122.1 (2)	C2—C6—H61	108.7
N2—C2—C6	114.4 (2)	C2—C6—H62	108.7
C3—C2—C6	123.5 (2)	C7—C6—H61	108.7
F1—C3—C2	121.0 (2)	C7—C6—H62	108.7
F1—C3—C4	115.4 (2)	H61—C6—H62	109.5
C2—C3—C4	123.6 (2)	C6—C7—H71	109.5
O2—C4—N1	121.3 (2)	C6—C7—H72	109.5
O2—C4—C3	126.6 (2)	C6—C7—H73	109.5
N1—C4—C3	112.1 (2)	H71—C7—H72	109.5
C2—C6—C7	112.5 (2)	H71—C7—H73	109.5
C1—N1—H1	118.5	H72—C7—H73	109.5
C4—N1—H1	118.5		
			
C5—O1—C1—N1	−179.1 (2)	N2—C2—C3—F1	178.6 (2)
C5—O1—C1—N2	1.4 (3)	N2—C2—C3—C4	−2.4 (4)
C1—N1—C4—O2	179.0 (2)	N2—C2—C6—C7	72.7 (3)
C1—N1—C4—C3	−0.0 (3)	C3—C2—C6—C7	−105.8 (3)
C4—N1—C1—O1	179.3 (2)	C6—C2—C3—F1	−3.0 (4)
C4—N1—C1—N2	−1.2 (4)	C6—C2—C3—C4	176.0 (3)
C1—N2—C2—C3	1.1 (4)	F1—C3—C4—O2	1.9 (4)
C1—N2—C2—C6	−177.5 (2)	F1—C3—C4—N1	−179.2 (2)
C2—N2—C1—O1	−179.9 (2)	C2—C3—C4—O2	−177.2 (3)
C2—N2—C1—N1	0.6 (4)	C2—C3—C4—N1	1.8 (4)

### Hydrogen-bond geometry (Å, °)

**Table:**

*D*—H···*A*	*D*—H	H···*A*	*D*···*A*	*D*—H···*A*
N1—H1···O2^i^	0.86	1.91	2.763 (2)	174

Symmetry codes: (i) −*x*+1, −*y*+1, −*z*+1.
